# An Incidental Tornwaldt Cyst Finding on the Postoperative Assessment of a Nasal Septum Deviation: A Case Report

**DOI:** 10.7759/cureus.39606

**Published:** 2023-05-28

**Authors:** Mustafa O Canatan, Mehmed F Canatan, Ahmed N Canatan

**Affiliations:** 1 Faculty of Medicine, Near East University, Nicosia, CYP; 2 Faculty of Medicine, Gaziantep University, Gaziantep, TUR; 3 Faculty of Medicine, Marmara University, Istanbul, TUR

**Keywords:** tornwaldt bursa, thornwald cyst, nasopharyngeal cyst, thornwaldt cyst, otorhinolaryngology, lack of intervention, asymptomatic, incidental finding, tornwaldt cyst

## Abstract

Tornwaldt cysts are rare, benign lesions that typically reside along the posterior wall of the nasopharynx. They are often discovered incidentally during routine imaging studies and can pose a diagnostic challenge due to their asymptomatic nature. This case report presents the incidental finding of a Tornwaldt cyst on a CT in an asymptomatic patient and highlights the lack of intervention required. The patient, a 28-year-old male, underwent a postoperative CT scan following septoplasty for a nasal septum deviation, revealing a well-defined cystic lesion in the midline of the nasopharynx consistent with a Tornwaldt cyst. Despite the presence of the cyst, the patient did not exhibit any associated symptoms such as nasal obstruction, headache, or recurrent infections. This case emphasizes the importance of recognizing and differentiating Tornwaldt cysts from other pathologies, as misdiagnosis and unnecessary interventions can lead to potential complications. Asymptomatic Tornwaldt cysts generally do not necessitate active intervention, but ongoing vigilance and individualized patient care remain vital to ensure optimal outcomes.

## Introduction

A Tornwaldt cyst is a rare benign cystic remnant of the notochord located along the midline of the nasopharynx [1]. These cysts are typically found in an incidental manner due to their small size and asymptomatic nature [2]. An incidence of 0.2% to 5% has been noted on routine brain and cervical MRI scans, as well as an incidence of 4% on postmortem autopsies [2-4]. Tornwaldt cysts do not have a sex preference but are usually found in patients between the ages of 15 and 30 [2]. Larger cysts give rise to a plethora of symptoms including but not limited to nasal obstruction, headache, postnasal discharge, neck stiffness, halitosis, foreign body sensation in the nasopharynx, and eustachian tube dysfunction-related otitis media [5]. Nasopharyngeal trauma as well as recurrent nasopharyngeal infections has been identified as common causes of Tornwaldt cysts [2]. Intervention is not necessary for asymptomatic patients; however, marsupialization is recommended as a surgical intervention in symptomatic patients [6]. Herein, we report a case of an incidental Tornwaldt cyst finding on a postoperative CT scan of a patient after undergoing septoplasty for a nasal septum deviation.

## Case presentation

**Figure 1:**
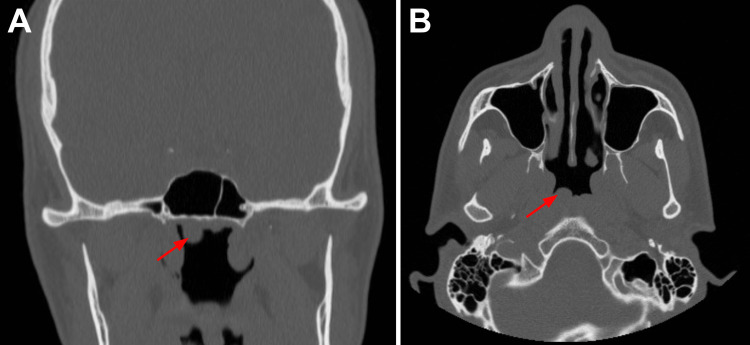
Postoperative CT scans of the Tornwaldt cyst Coronal (A) and transverse (B) views of CT scans showing a solitary Tornwaldt cyst (red arrow), 8 mm in diameter, along the posterior wall of the nasopharynx

A 28-year-old male patient presents to the ENT outpatient clinic for a second opinion on a Tornwaldt cyst he himself identified on an old, four-year-old CT scan. The patient is a medical doctor himself. He states that he visited a plastic and reconstructive surgery clinic four years ago for evaluation of a trauma-related nasal septum deviation. The patient recounts encountering a head-on collision while running with one of his friends in elementary school. Ever since the collision, the patient had experienced difficulty breathing due to obstruction in his nostrils. The patient claims that his nose had a mildly crooked appearance from the outside which was part of the reason why he wanted to undergo surgery. The patient was diagnosed with a deviated septum, and septoplasty was performed four years ago. The surgery was successful and the patient no longer has difficulty breathing. The patient recently graduated from medical school and was going through his old personal medical files when he came across his CT scans from four years ago (Figure 1). The CT report from four years prior states that a solitary 8 mm cyst was observed to the right of the midline of the posterior wall of the nasopharynx (Tornwaldt cyst). His previous surgeon had not informed him about the presence of these cysts which is what prompted him to present to the ENT clinic for evaluation.

**Figure 2:**
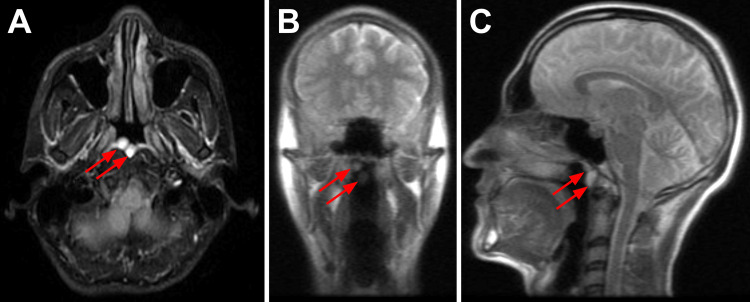
T2-weighted and diffusion-weighted MRI scans of the Tornwaldt cysts T2-weighted fat-suppressed fluid-attenuated inversion recovery image transverse view (A), diffusion-weighted imaging sequences of coronal (B) and sagittal (C) views of magnetic resonance imaging scans showing two hyperintense Tornwaldt cysts (red arrows) each with a diameter of 8 mm, along the posterior wall of the nasopharynx

MRI was performed, now revealing two hyperintense appearing Tornwaldt cysts, each 8 mm in diameter (Figure 2). The old cyst seen on the CT scan from four years ago remains in its position on the posterior wall of the nasopharynx. A second cyst, not previously seen on the CT scan, is located medial to the former Tornwaldt cyst, directly along the midline of the posterior wall of the nasopharynx.

The patient is asymptomatic and otherwise healthy; therefore, no intervention of any sort is recommended. The patient is informed that the cysts are benign lesions that he does not have to worry about and that they may regress by themselves. The patient is also informed that the Tornwaldt cysts may turn symptomatic if they continue to grow, in which case surgical interventions such as excision or marsupialization would be necessary for symptomatic relief.

## Discussion

Tornwaldt cysts (also known as Thornwaldt, Thornwald, or nasopharyngeal cysts) are rare benign cystic remnants of the notochord that are located along the midline of the posterior wall of the nasopharynx [1]. The cyst was first identified in 1840 by Mayer August Franz Joseph Carl in autopsy specimens and later demonstrated as a pathological manifestation in 1885 by Gustavus Ludwig Tornwaldt [1]. Tornwaldt cysts are typically greater than 7 mm in diameter and are characterized by the absence of inflammation in neighboring soft tissues [2]. Chemoradiation, as well as surgical trauma acquired during adenoidectomies has been identified as etiologic factors in the development of these cysts [3]. Tornwaldt cysts are classified either as cystic or crusting depending on the pattern of drainage [3]. The drainage pathway of cystic types is often occluded resulting in the formation of a cyst, whereas crusting types directly drain into the nasopharynx without obstruction thereby preventing cyst formation [3]. An incidence of 0.2% to 5% has been noted on routine brain and cervical MRI scans, as well as an incidence of 4% on postmortem autopsies [2-4]. Tornwaldt cysts are present in both males and females equally but are most commonly identified in patients between the ages of 15 and 30 [2]. Tornwaldt cysts are typically found in an incidental manner due to their small size and asymptomatic nature and, therefore, often remain undiagnosed for decades [2]. The cysts may enlarge with time and give rise to numerous symptoms including nasal obstruction, headache, postnasal discharge, neck stiffness, seizures, halitosis, foreign body sensation in the nasopharynx, and otitis media [5]. Tornwaldt cysts may easily be appreciated through naso-endoscopy as encapsulated lesions along the midline of the posterior wall of the nasopharynx; however, MRI remains the gold standard for diagnosis [7]. Hyperintense lesions may be appreciated on T1-weighted and T2-weighted MRI scans due to associated hemorrhage, fluid, or protein accumulation within the cysts [2]. CT scans are also useful for diagnosis and display low-density, well-circumscribed lesions along the midline of the posterior wall of the nasopharynx [8]. Adenoid retention cyst, Rathke’s cleft cyst, retropharyngeal abscess, pharyngeal polyp, and branchial cleft cyst should all be included in the differential diagnosis of a suspected Tornwaldt cyst [7]. Adenoid retention cysts are known to cause nasal obstruction and can easily be identified near the adenoids, whereas retropharyngeal abscesses are accompanied by infections and can be found in the retropharyngeal space with surrounding inflammation [7]. Rathke's cleft cysts are typically found in the sellar or suprasellar region with a thin enhancing rim, whereas branchial cleft cysts are typically located along the anterior border of the sternocleidomastoid muscle [7]. Treatment is not necessary for asymptomatic patients; however, excision or marsupialization is recommended as a surgical intervention in symptomatic patients [6]. Symptomatic Thornwaldt cyst cases can safely undergo surgical treatment using the transnasal endoscopic marsupialization technique due to its convenient implementation, brief surgical duration, minimal recurrence rates, and effective therapeutic outcomes [6]. Aspiration of the cyst alone is not recommended as the rate of recurrence is high; however, the majority of patients that undergo surgical excision or marsupialization end up with a complete resolution of symptoms alongside lower rates of recurrence [1,2].

## Conclusions

Tornwaldt cysts are typically benign, congenital lesions located along the midline of the posterior wall of the nasopharynx and are often discovered incidentally during routine imaging studies. The absence of symptoms in this patient further supports the notion that asymptomatic Tornwaldt cysts generally do not require active treatment or intervention. However, it is crucial for clinicians to be aware of these cysts and differentiate them from other pathologies, as misdiagnosis or unnecessary interventions can lead to potential complications. Patients incidentally found to have asymptomatic cysts should be informed about the benign nature of the disease and any complications that may arise in the event of the cyst growing. Continued vigilance and appropriate management based on the patient's individual clinical presentation remain essential for optimal patient care.
